# Expression and potential roles of sodium-potassium ATPase and E-cadherin in human gastric adenocarcinoma

**DOI:** 10.1371/journal.pone.0183692

**Published:** 2017-08-23

**Authors:** Shih-Ho Wang, Kuan-Lin Wang, Wen-Kai Yang, Tsung-Han Lee, Wan-Yu Lo, Jane-Dar Lee

**Affiliations:** 1 Division of General Surgery, Department of Surgery, Kaohsiung Chang Gung Memorial Hospital and Chang Gung University, College of Medicine, Kaohsiung, Taiwan, R.O.C; 2 Center for General Education, Cheng Shiu University, Kaohsiung, Taiwan, R.O.C; 3 Department of Life Sciences, National Chung Hsing University, Taichung, Taiwan, R.O.C; 4 Bachelor Degree Program in Animal Healthcare, Hungkuang University, Taichung, Taiwan, R.O.C; 5 Department of Biotechnology, Hungkuang University, Taichung, Taiwan, R.O.C; 6 Department of Urology, Feng-Yuan Hospital, Ministry of Health and Welfare, Taichung, Taiwan, R.O.C; 7 Central Taiwan University of Science and Technology, Taichung, Taiwan, R.O.C; 8 Department of Surgery, Taichung Armed Forces General Hospital, Taichung, Taiwan, R.O.C; National Cancer Center, JAPAN

## Abstract

**Background:**

Gastric adenocarcinoma originates from an abnormal epithelium. The aim of this study was to investigate the expression of sodium-potassium ATPase (NKA), a transmembrane protein located in the epithelium for Na^+^ and K^+^ transportation, and E-cadherin, which are both crucial for the epithelium and adherens junction, as potential gastric cancer biomarkers.

**Methods:**

45 patients diagnosed with gastric adenocarcinoma were recruited. Immunohistochemistry and immunofluorescence were conducted to for localization of NKA α1-, β1-isoform, and E-cadherin. NKA enzyme activity was determined by NADH-linked methods and immunoblotting of NKA α1-, β1-isoform, and E-cadherin were performed to evaluate protein expression.

**Results:**

Immunostaining revealed that NKA was co-localized with E-cadherin in the glands of the gastric epithelium. Both NKA activity and α1-isoform protein expression were reduced in the study group (P < 0.05), indicating impaired NKA functions. In the adherens junctions, the NKA β1-isoform and E-cadherin were significantly reduced in the study groups (P < 0.05), indicating the adhesion force between cells may have been weakened.

**Conclusions:**

A significant decrease in NKA function (protein and activity) and E-cadherin in tumor lesions appear promising biomarker for gastric adenocarcinoma. Therefore, developing screening methods for detecting NKA function may be beneficial for the early diagnosis of gastric cancer. In our knowledge, this study was the first to investigate the NKA and E-cadherin expression in the relation of gastric adenocarcinoma in human patients.

## Introduction

Gastric adenocarcinoma developed from epithelial mucosa and gland cells [1] is a very common cancer in Asia because of the dietary habits and lifestyle of Asian people. Because most patients do not show early symptoms, early detection and an understanding of tissue morphology and pathology within the adeno glands may help in the early diagnosis of this disease. However, there are no adequate biomarkers for the early screening of gastric cancer.

In the human epithelium, sodium-potassium ATPase (NKA) is a ubiquitous membrane-bound protein that is essential for cell polarity; NKA actively maintains the Na^+^ and K^+^ gradients across the plasma membrane to maintain ion concentrations [2]. NKA consists of an (αβ)_2_ protein complex, which contains four catalytic α (α1–4) and three glycosylated β (β1–3) isoforms [3]. Among the possible combinations of the two subunits, α1β1 is widely expressed and appears to be the major form within epithelial tissues [4]. Many studies have investigated the potential roles of NKA in certain cancers [5]. Abnormal NKA expression and activity have been implicated in the development and progression of different cancers [6].

The NKA β1-isoform functions as a type of adherens junctions by forming β1-β1 bridges between cells. Adhesive forces prevent animal tissues from dissociating into their component cells [7]. In medulloblastoma, a lack of the NKA β1-isoform results in an increased invasion and migration rate [8]. Furthermore, Barwe et al. [9] reported that E-cadherin helps NKA β1 maintain cell organization and structure and stabilizes cell polarity. E-cadherin is also a major protein in adherens junctions [10] and has been widely studied in some cancers [11].

NKA and E-cadherin were reported to be crucial in the epithelium. Thus, the present study examined their changes between control (normal) and study (tumor) groups. The results of this study reveal a promising, distinct, and specific biomarker for gastric adenocarcinoma at the protein level.

## Materials and methods

### Experimental samples

The protocol used for sampling was reviewed and approved by the Institutional Review Board of Kaohsiung Chang Gung Memorial Hospital with the approval number 103-1279B. Stomach specimens were obtained by surgery from 45 patients (20 females and 25 males) diagnosis with gastric adenocarcinoma and 60% of patients were *Helicobacter pylori* infected. These tissues, which were at least 5 cm apart from the tumor margin and normal tissues, were divided into two groups—the study (tumor) and the control groups. After resection, the samples were immediately fixed with 10% formalin or stored at -80°C until analysis. The samples were then classified and categorized as stage I–IV according to the seventh edition of the American Joint Cancer Committee/Union for International Cancer Control tumor-node-metastasis classification. All patient details are listed in Table 1.

**Table 1:** Characteristics of enrolled patients with gastric adenocarcinoma.

Group	N	Sex (♂: ♀)	Age (years)^†^	Blood CEA (ng/mL) ^†^
Total	45	25:20	68.47 ± 1.61 [44–90]	4.10 ± 0.71 [0.6–21.5]
Stage				
I	10	6:4	71.4 ± 2.78 [59–80]	3.66 ± 1.37 [1.21–2.84]
II	10	8:2	69.75 ± 4.47 [44–84]	4.85 ± 2.11 [0.60–21.50]
III	20	15:5	66.83 ± 2.26 [49–90]	3.59 ± 0.73 [0.64–10.05]
IV	5	5:0	70.4 ± 4.57 [62–87]	8.95 ± 2.92 [2.07–15.00]

^†^, Mean ± S.E.M. [Range]

CEA, carcinoembryonic antigen; N, sample size.

### Paraffin sectioning and immunostaining

Paraffin sectioning and immunostaining were conducted as described previously with some modifications [12, 13]. After fixation, the samples were dehydrated and embedded into paraffin blocks. The samples were cut into 4-μm thick sections. The paraffin sections were deparaffinized and then pre-incubated in 10 mM sodium citrate at 100°C for 15 min, 3% peroxide for 10 min, and 5% bovine serum albumin for 20 min.

For immunohistochemical (IHC) staining, the slides were stained with primary antibody (Table 2) or negative control (phosphate-buffered saline, PBS) followed by analysis with a commercial kit (PicTure^TM^; Zymed, South San Francisco, CA, USA). Finally, the sections were counterstained with hematoxylin (Cat. No.1.05175.0500; Merck, Darmstadt, Germany) and rinsed with tap water.

**Table 2:** Detailed information of antibodies used in this study.

Antibody	Company	Catalog	Host	Origin	App.	Dilution
Primary						
NKA α1	DSHB	a6F	MM	Avian NKA α1	IB	1000×
	Abcam	ab76020	RM	Human NKA α1	IHC	200×
					IF	200×
NKA β1	Abcam	ab2873	MM	Sheep NKA β1	IB	2000×
					IF	200×
E-cadherin	Invitrogen	33–4000	MM	Human E-cadherin	IB	1000×
					IF	200×
GAPDH	Santa Cruz	sc-25778	RP	Human GAPDH	IB	10000×
Secondary						
488-Rabbit	Jackson	111-507-003	Goat	N/A	IF	400×
549-Mouse	ABM	SD010	Goat	N/A	IF	400×
HRP-Rabbit	Genetex	GTX213110-01	Goat	N/A	IB	5000×
HRP-Mouse	Genetex	GTX213112-01	Goat	N/A	IB	5000×

ABM, Applied Biological Material Inc.; App., application; DSHB, Developmental Studies Hybridoma Bank; GAPDH; glyceraldehyde 3-phosphate dehydrogenase; HRP, horseradish-peroxidase-conjugated; IB, immunoblotting; IF, immunofluorescence staining; IHC, immunohistochemistry; MM, mouse monoclone; NKA, sodium-potassium ATPase; RM, rabbit monoclone; RP, rabbit polyclone; N/A, not applicable.

For double immunofluorescence (IF) staining, after pre-incubation the slides were stained with primary antibodies (Table 2) at room temperature for 2 h. The slides were treated with secondary antibodies (Table 2) for 1 h followed by three washes with PBS. Next, the slides were incubated with different primary antibodies overnight, and then incubated with the appropriate secondary antibodies. Finally, the sections were mounted using Dapi Fluoromount-G (SouthernBiotech, Birmingham, AL, USA).

The immunoreactions were observed under a microscope (BX50; Olympus, Tokyo, Japan) with a cooling-charge-coupled-device camera (DP72; Olympus) and analyzed using CellSens standard version 1.4 software (Olympus).

### Preparation of tissue homogenates

Tissue homogenates were prepared by using the methods of Lee et al. [13] with few modifications. Human stomach epithelia were cut to 5 mm^3^ and soaked in 500 μL commercial Cell Culture Lysis 5x Reagent (E1531; Promega, Madison, WI, USA) containing protease inhibitors (11836145001; Roche, Basel, Switzerland) for 1 min in a 2-mL microtube, and then homogenized with a Polytron PT1200E (Kinematica, Lucerne, Switzerland) at maximum speed for 20 s on ice. Homogenates were then centrifuged at 5500 ×*g* and 4°C for 5 min. The supernatants were collected and used immediately for the NKA activity assay or stored at -80°C for protein concentration measurements or immunoblotting. The protein concentrations were determined using the BCA Protein Assay (Pierce, Rockford, IL, USA) with bovine serum albumin as a standard.

### NKA activity assay

The activity was determined according to the NADH-linked methods [13, 14]. ADP derived from the hydrolysis of ATP by ATPase was enzymatically coupled to the oxidation of reduced NADH using pyruvate kinase and lactate dehydrogenase. Before the assay, a standard curve was determined from 0 to 30 nmol ADP per well at 340 nm at 37°C after adding 200 μL assay mixture (50 mM imidazole, 5 mM ATP, 10 mM phosphoenolypyruvate, 0.25 mM NADH, 1.1 U lactate dehydrogenase, 0.9 U pyruvate kinase, 100 mM NaCl, 8 mM MgCl_2_∙ 6H_2_O, 20 mM KCl, pH 7.5) for at least 5 min in a VERSA max microplate reader (Molecular Devices, Sunnyvale, CA, USA). The slope of the standard curve should be -0.012 to -0.015 absorbance units nmol/ADP. A 10-μL sample was loaded in a well, and 200-μL assay mixture was added with or without 1 mM ouabain (a specific NKA inhibitor; O3125; Sigma, St. Louis, MO, USA); each sample was assayed in triplicate. Absorbance was measured every 15 s for up to 20 min in the microplate reader at 340 nm and 37°C. The linear rate from 600 to 900 s for each pair of triplicate well was determined. The NKA activity was calculated as the difference in slope of ATP hydrolysis (i.e., NADH reduction) in the presence and absence of ouabain, and was expressed as μmol ADP per mg protein per hour.

### Immunoblotting

Immunoblotting procedures were modified from Lee et al. [13, 15] with a few modifications. Aliquots containing 20 μg of sample homogenates were heated with denaturing buffer at 60°C for 15 min. The samples were separated by electrophoresis on 10% sodium dodecyl sulfate polyacrylamide gels. The separated proteins were transferred to 0.45 μm polyvinylidene fluoride membranes (Millipore, Bedford, MA, USA) using a tank transfer system (Mini protean 3; Bio-Rad, Hercules, CA, USA). The membranes were pre-incubated for 2 h in PBS containing 0.2% (v/v) Tween 20 and 5% (w/v) nonfat dried milk to minimize nonspecific binding. Next, the blots were incubated at 4°C overnight with antibodies (Table 2). The membranes were then incubated at room temperature for 1 h with secondary antibodies (Table 2). Blots were developed using Immobilon^TW^ Western (Millipore). Signals were obtained using the Chemidoc XRS+ image system (Bio-Rad), and then the data were analyzed with Image Lab software (version 3.0; Bio-Rad). The results were converted to numerical values to compare the relative protein abundance of the immunoreactive bands.

### Statistical analysis

All analyses including NKA activity and protein expression from immunoblotting were sorted into control and study groups with or without stages (Table 1). Values were shown as the mean ± S.E.M. (standard error of the mean). Statistical significance was compared and graphed using Graphpad software (version 6; La Jolla, CA, USA) with two tailed *t*-test and passed normal distribution. The significance level was set at *P* < 0.05.

## Results

### Localization of NKA and E-cadherin on gastric epithelium

**Fig 1:**
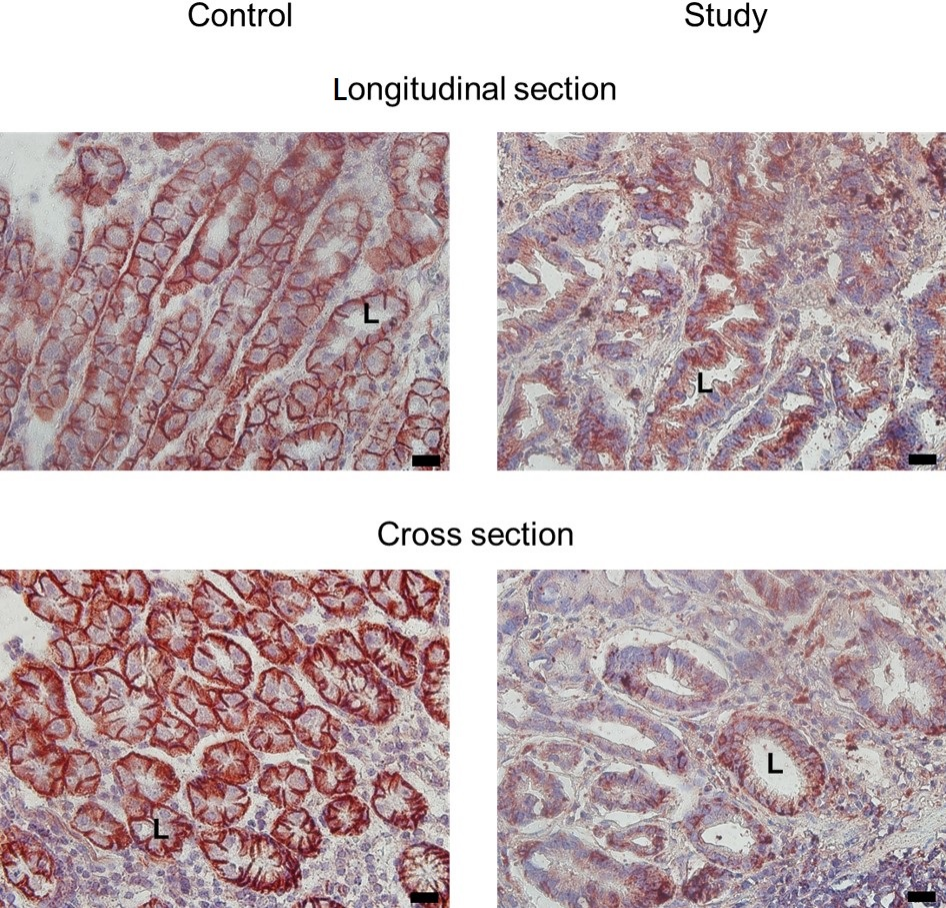
Immunohistochemical NKA staining of human gastric samples of the control and study groups. NKA shown in red color. L, lumen. Scale bar: 20 μm.

**Fig 2:**
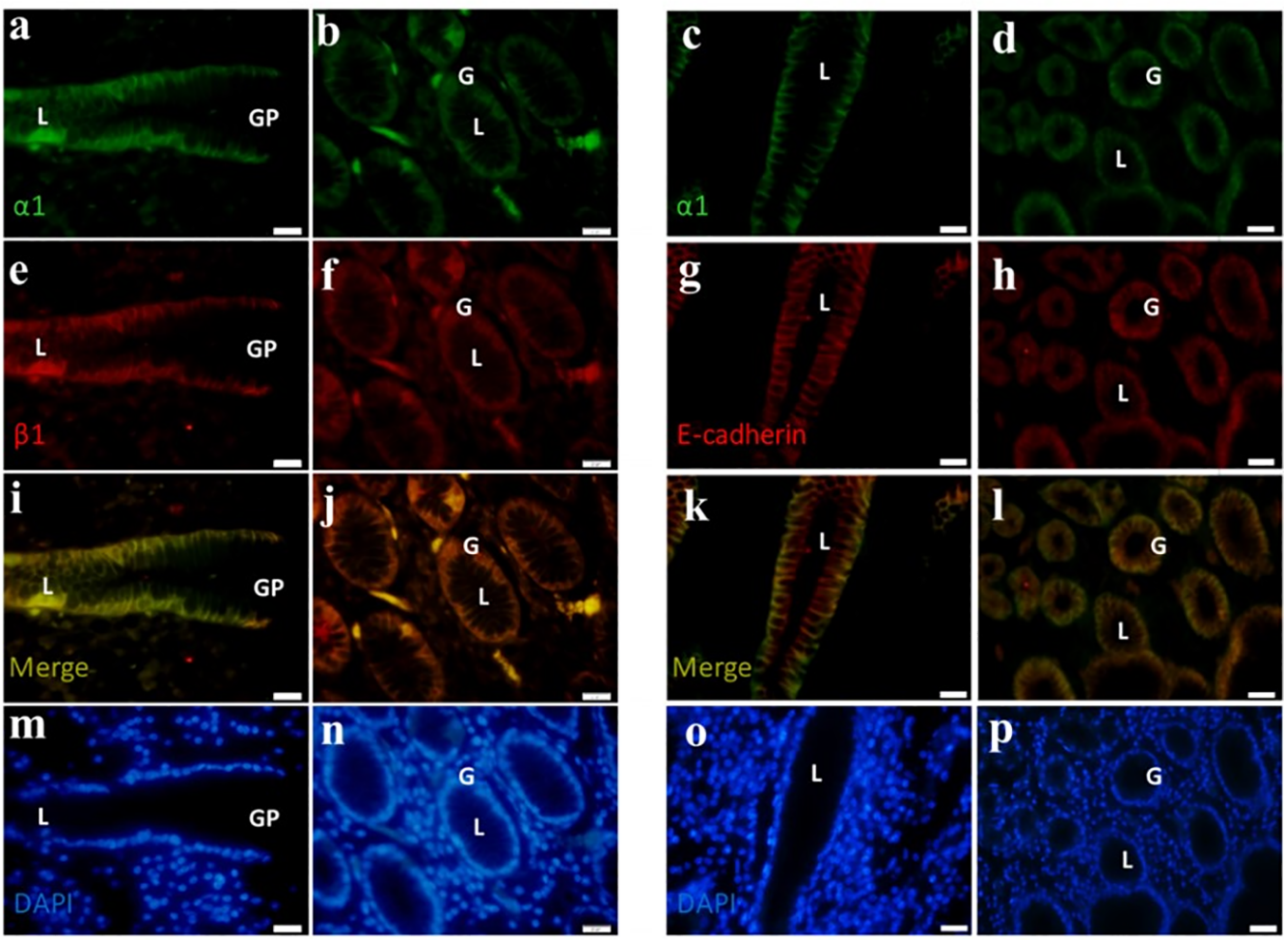
**a–p Multiple immunofluorescent staining of NKA α1- (green; a, b, c, d), β1-isoforms (red; e, f), and E-cadherin (red; g, h) in the control group.** (a, c, e, g, i, k) Longitudinal section of gastric pit (GP) and epithelium; (b, d, f, h, j, l) cross-section of fundic gland. Merged (yellow; i–l) and DAPI (blue; m–p) images were used to reveal the co-localization of α1-isoform with β1-isoform and with E-cadherin compared to the cell nuclei (blue). G, gland; L, lumen. Scale bar = 20 μm.

IHC staining of the tissues with NKA α1 antibody revealed that the NKA α1-isoform (red color) was distributed on the gastric epithelial cells. In the longitudinal and cross-sections (Fig 1), NKA α1 was detected in the basolateral membrane of gland cells, where the cellular apical regions and lumens exhibited low or no signal. In gastric adenocarcinoma, tissue structures were fragmented and NKA α1 was expressed poorly within the gland cells. In contrast, in IF staining of the paraffin sections, the immunosignals of the NKA α1- and β1-isoforms were distinguished as green and red, respectively (Fig 2A, 2B, 2E and 2F). Co-localization of the NKA α1- and β1-isoforms was shown in yellow in the merged images (Fig 2I and 2J), with DAPI counterstaining of the nucleus shown in blue (Fig 2M and 2N). In addition, the immunosignals of NKA α1 and E-cadherin were green and red, respectively (Fig 2C, 2D, 2G and 2H). Co-localization of NKA α1 and E-cadherin was shown in yellow in the merged images (Fig 2K and 2L), with DAPI counterstaining of the nucleus shown in blue (Fig 2O and 2P). These results demonstrate that the NKA complex with E-cadherin was expressed on the basolateral regions of tissues and close to the base region. The results of IHC staining revealed the localization of NKA on the gastric tissue (Fig 1), and IF confirmed the co-localization of NKA α1 and β1 with E-cadherin (Fig 2). In addition, compared to the control group, in the study group the histological structures appeared loose and exhibited larger lumens of the glands.

### Protein expression in control and study groups

**Fig 3:**
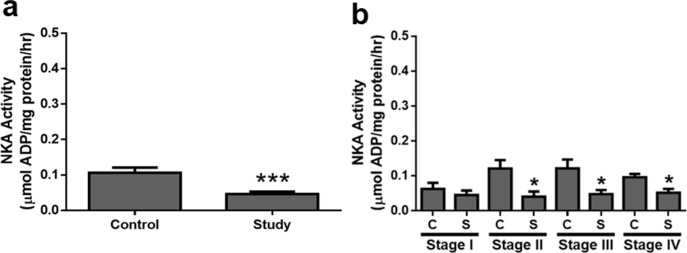
**a–b NKA activity of gastric epithelium in the (a) control and study groups, and (b) sorted by stages.** The asterisks indicate a significant difference between the control (C) and study (S) groups. Values are expressed as the means ± SEM. *, P < 0.05; ***, P < 0.001.

**Fig 4:**
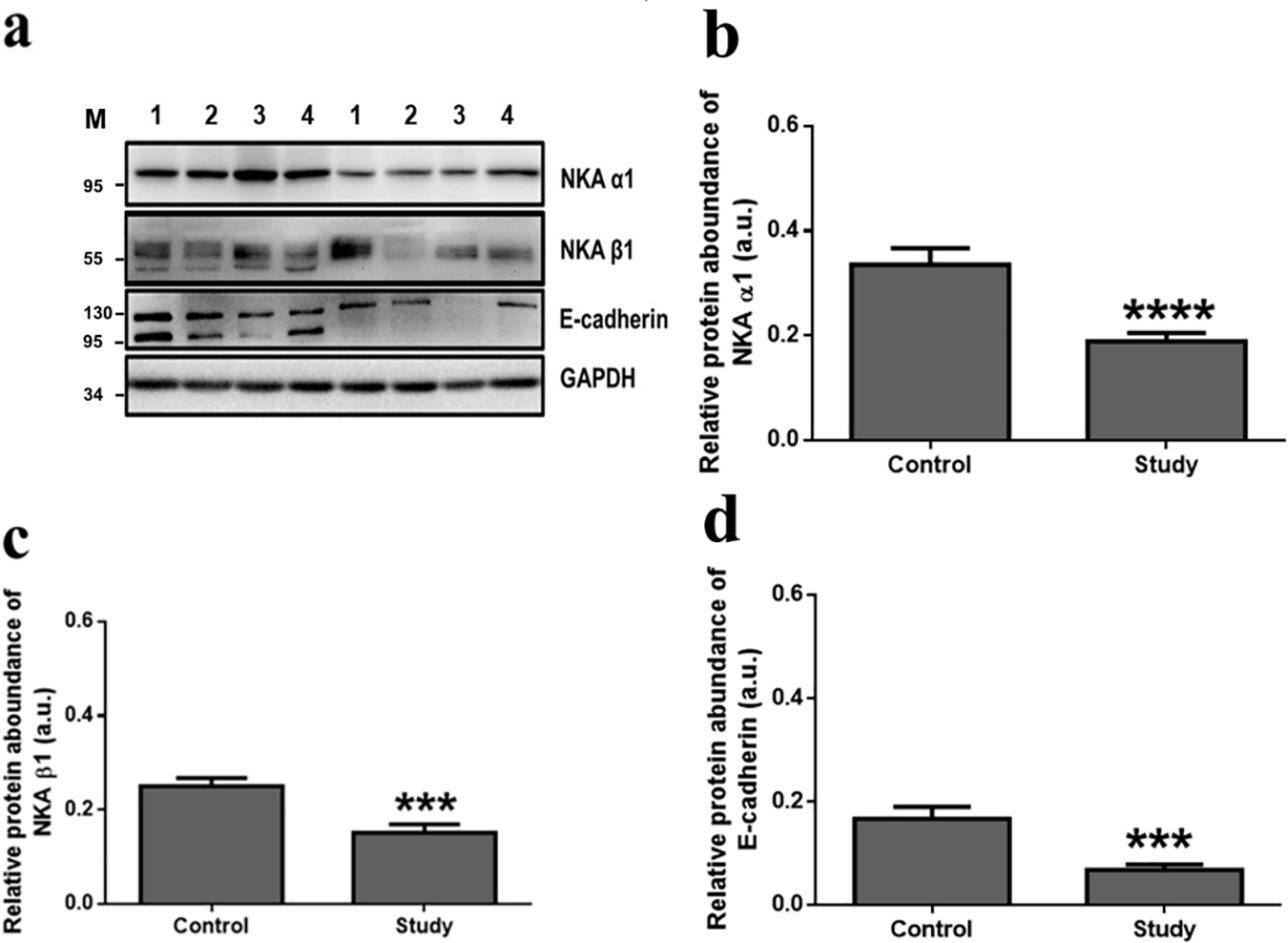
**a–d Immunoblotting of different proteins in control and study groups of gastric adenocarcinoma.** (a) Representative immunoblots of different target proteins in gastric epithelium of the control and study groups (from patients 1 to 4) with GAPDH as a loading control. M, marker. Relative protein abundance of NKA α1 (b), NKA β1 (c), and E-cadherin (d) in gastric epithelium of the control and study groups. The asterisks indicate a significant difference between the control and study groups. Values were expressed as the means ± SEM. A.u., arbitrary unit. ***, P < 0.001; ****, P < 0.0001.

**Fig 5:**
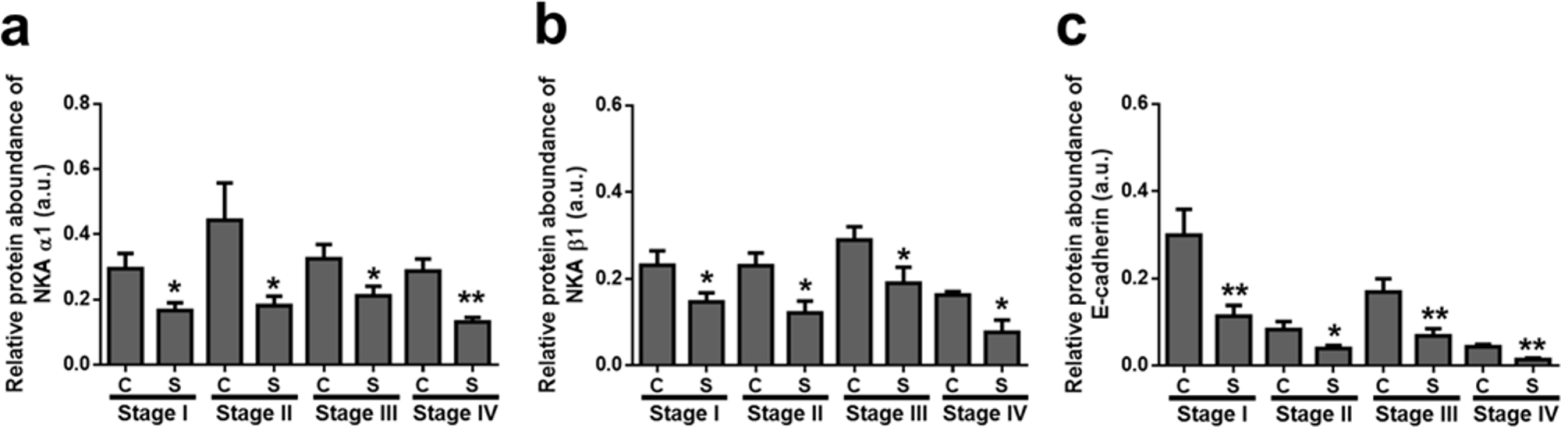
**a–c Immunoblotting of different proteins in control and study groups with stages.** NKA α1 (a), NKA β1 (b), and E-cadherin (c) in gastric epithelium of the control (C) and study (S) groups sorted by stage I–IV. The asterisks indicate a significant difference between the control and study groups. Values were expressed as the means ± SEM. A.u., arbitrary unit. *, P < 0.05; **, P < 0.01.

NKA enzyme activity was determined in the coupled assay and a significant difference was detected between the control and study groups with stage sorting (Fig 3A and 3B). NKA activity decreased in the study groups and subjects with stage II–IV showed impaired functions of NKA such as ion transport. In contrast, representative immunoblots of the NKA α1-isoform are shown in Fig 4A, and protein expression in the control and study groups were significantly different, as shown in Fig 4B (P < 0.0001). Interestingly, reduction of the NKA α1-isoform was detected in all stages (Fig 5A). In addition, representative immunoblots of two adherens proteins, the NKA β1-isoform and E-cadherin, are shown in Fig 4A. In the study groups, the amounts of both proteins were decreased compared to in the control groups (Fig 4C and 4D) and different stages (Fig 5B and 5C). Taken together, the expression of the proteins evaluated decreased in the study group.

## Discussion

In the epithelium, NKA plays a key role in maintaining homeostasis and tightening cell polarity [2, 3]. NKA activity is also required for adherens junction force [16]. A decreased of NKA activity indicates the reduced of ion exchange ability, which is essential for basic fundamental cellular functions [6]. In this study, the NKA α1-isoform was mainly localized to the basolateral regions of gastric epithelial cells (Fig 1), similar to the NKA distribution in other human tissues and organs [17]. A weaker NKA signal and looser structure of the gland tissues were observed in the tumor group. Similarly, NKA activity and protein expression of the NKA α1-isoform were decreased in gastric adenocarcinoma. Moreover, for NKA activity, subjects in stage II–IV showed decreased enzyme activity, indicating that more advanced tumor stages will show lower NKA function. Protein expression of the NKA α1-isoform was significantly different at each stage in the study groups, indicating that factors of carcinogenesis in all stages, such as tumor size and metastasis [18], may alter NKA function in gastric adenocarcinoma.

In other tumors, reduced NKA function (mRNA, protein, and activity) was also reported [8, 19]. The decrease of NKA function may induce tumor metastasis and migration. The lack of NKA activity indicates impaired ion exchange, which leads to a decrease in fundamental cellular functions [6]. These results revealed lower NKA activity and α-subunits (the catalytic subunit) were in epithelial cells of gastric adenocarcinoma and indicate that the homeostasis of the gastric epithelium was affected, leading to numerous physiological changes such as tumor progression [20].

In the adherens junction, the relative NKA β1-isoform and E-cadherin protein expression were also decreased significantly in the study groups compared that of the control groups (Fig 4C and 4D). Similar results were observed in gastric and breast cancer in which E-cadherin expression was decreased [21, 22]. Dysregulation of E-cadherin may lead to gastric epithelial cell dysfunction and contribute to gastric cancer development during the progression to tumor malignancy [23]. In addition, E-cadherin was expressed with the NKA α1-isoform (Fig 2K and 2L), revealing that E-cadherin localized to the basolateral regions of the gland cells. By staging, decreased expression of NKA β1 and E-cadherin (Fig 5B and 5C) indicated a weaker force in epithelium at all different stages. NKA β1 interacts with E-cadherin to maintain cellular structure [24–26]. Therefore, these results revealed an impaired function of the cell-cell adherens force in gland epithelial cells of the gastric adenocarcinoma, suggesting that tumor migration may occur because of the lack of a binding force from the cell properties [11]. However, the weakened adherens proteins (i.e. NKA β1 and E-cadherin) were also observed in the control groups, indicating that normal tissue may already be affected by tumor cells without any positive tumor signs

In conclusion, in gastric adenocarcinoma, reduced NKA activity and decrease in levels of NKA α1-, β1-isoforms, and E-cadherin were observed. These results indicate that impaired NKA function in maintaining cellular homeostasis and epithelial junctions for sustaining the cell structures results in more rapid tumor metastasis. These significant changes in NKA and E-cadherin in different tumor stage are a promising biomarker for gastric adenocarcinoma. To our knowledge, this is the first report of decreased expression of NKA and E-cadherin in gastric adenocarcinoma. Our results reveal that the levels of these proteins can be used as potential biomarkers for the early detection of human gastric cancer.
